# The Effects of Food Nutrients and Bioactive Compounds on the Gut Microbiota: A Comprehensive Review

**DOI:** 10.3390/foods13091345

**Published:** 2024-04-26

**Authors:** Yijun Zheng, Chunyin Qin, Mingchun Wen, Liang Zhang, Weinan Wang

**Affiliations:** 1 Clinical Pharmacy (Sino-Foreign Cooperation) Class, School of Chinese Materia Medica, Tianjin University of Traditional Chinese Medicine, Tianjin 301617, China; zhengyijun0620@foxmail.com; 2 State Key Laboratory of Tea Plant Biology and Utilization, Anhui Agricultural University, 130 Changjiang West Road, Hefei 230036, China; qin_chunyin@163.com (C.Q.); 22710050@stu.ahau.edu.cn (M.W.); 3 Guangdong Key Laboratory for Research and Development of Natural Drugs, School of Pharmacy, Guangdong Medical University, No. 1 Xincheng Blvd, Dongguan 523808, China

**Keywords:** gut microbiota, dietary fiber, polyphenols, protein, bioactive compounds

## Abstract

It is now widely recognized that gut microbiota plays a critical role not only in the development and progression of diseases, but also in its susceptibility to dietary patterns, food composition, and nutritional intake. In this comprehensive review, we have compiled the latest findings on the effects of food nutrients and bioactive compounds on the gut microbiota. The research indicates that certain components, such as unsaturated fatty acids, dietary fiber, and protein have a significant impact on the composition of bile salts and short-chain fatty acids through catabolic processes, thereby influencing the gut microbiota. Additionally, these compounds also have an effect on the ratio of *Firmicutes* to *Bacteroides*, as well as the abundance of specific species like *Akkermansia muciniphila*. The gut microbiota has been found to play a role in altering the absorption and metabolism of nutrients, bioactive compounds, and drugs, adding another layer of complexity to the interaction between food and gut microbiota, which often requires long-term adaptation to yield substantial outcomes. In conclusion, understanding the relationship between food compounds and gut microbiota can offer valuable insights into the potential therapeutic applications of food and dietary interventions in various diseases and health conditions.

## 1. Introduction

“Gut microbiota”, encompassing a diverse array of bacteria, viruses, fungi, and protozoa, has emerged as a key focus of scientific inquiry, propelled by breakthroughs in metagenomics and next-generation sequencing technologies. These microbial communities fulfill critical functions, ranging from macronutrient metabolism and micro-nutrient synthesis to the detoxification of xenobiotics, bolstering of intestinal barrier integrity, and modulation of the host’s immune response. The gut microbiome’s composition is shaped by a confluence of environmental and genetic factors, with disruptions to its symbiotic equilibrium with the host potentially leading to the release of inflammatory mediators and compromise of intestinal barriers [1]. The gut microbiota has been the subject of much research in the life sciences because of its correlations with different disease phenotypes, physiological states, nutritional intake, and dietary phytochemical-based therapies. In the field of life sciences research, the gut microbiota is extensively studied for its associations with various disease phenotypes, physiological conditions, nutritional intake, and interventions involving food phytochemicals. This line of inquiry has highlighted the disparities in the relative abundance of specific microbial taxa, such as the enrichment of *Firmicutes* and *Actinobacteria* in obesity in comparison to Bacteroidetes’ predominance in lean individuals [2]. Using cutting-edge analytical methods like 16S rRNA sequencing and metagenomics, animal models have proven crucial in examining the diversity of the microbiota and how it dynamically changes in response to dietary treatments, going beyond research conducted on humans [3]. On the plant side, some phytochemicals have been shown to improve markers of disease risk without significant changes in the gut microbiota, for example, the phytosterol esters present in navy beans have positive impact on colon health in cancer survivors without changing gut microbiota composition [4].

The gut microbiota and its relationship with diseases, specifically obesity and leanness, were first investigated by analyzing the microbial content in human feces [1]. The analysis of obesity-associated gut microbiota revealed an enrichment of *Actinobacteria* and *Firmicutes*, while lean individuals showed a predominance of *Bacteroidetes* [1]. Many studies have focused on animal models to examine the diversity and composition changes of gut microorganisms using techniques such as 16S RNA or metagenome analysis [1]. In this review, we aim to summarize the impact of food compositions, such as carbohydrates, fatty acids, food proteins, and dietary phytochemicals, on the evolution of gut microbiota. We will explore how these food components can induce or interfere with changes in the gut microbial community. This review study addresses a significant vacuum in the existing research literature by delving into the influence of dietary components on the development of gut microbiota. Although prior studies have discussed the relationships between gut microbiota and health and illness, this article focuses on the precise processes of different dietary components in controlling gut microbial diversity and function, as well as how these mechanisms impact host health. In this context, we focus on the most recent study findings, such as the impact of carbohydrates, fatty acids, proteins, and dietary phytochemicals on gut microbiota. Comparable publications, such as Zsálig et al.’s review in 2023, which investigated the association between gut microbiota and obesity [5], and Puljiz Z et al.’s review in 2023, which investigated the effects of dietary fiber on gut microbial diversity [6], give useful information; however, they frequently focus on specific food components or health issues. Unlike these studies, this review seeks to give a broader viewpoint by investigating numerous dietary components and how they interact with gut bacteria, therefore influencing health. This study seeks to present a new and complete perspective on how gut microbes impact health through dietary components by merging the most recent research findings with traditional viewpoints.

## 2. The Effects of Nutrients on Microbiota and Diseases

### 2.1. The Effects of Probiotic Microorganisms and Mineral Ions

The term “microorganisms promoting the growth of other microorganisms” was first used to describe probiotic microbes [7]. In the modern sense, probiotics are live bacteria that help maintain a healthy balance of the autochthonous microbial community in the gastrointestinal tract (GT). Although they might not always reside in the GT, these bacteria ought to have a “…beneficial effect on the general and health status of man and animal” [8,9]. “Mono- or mixed cultures of live microorganisms which, when applied to animal or man, beneficially affect the host by improving the properties of the indigenous microflora” is the more exact definition of probiotics that has been given in recent years [10]. To enhance the wellbeing of both people and animals, probiotics are regarded as “viable preparations in foods or dietary supplements”. Probiotic microorganisms, particularly *lactic acid bacteria*, have been extensively studied for their role in promoting health and alleviating certain diseases. Probiotics are widely used in the food industry to produce fermented products and functional foods. They are also available as freeze-dried supplements. Maintaining a healthy gut microbiota is crucial for overall wellbeing [11]. Evidence-based investigations have revealed that probiotics can effectively prevent or treat infectious diarrhea, inflammatory bowel disease, and other intestinal diseases, by interfering with pathogens, improving intestinal barrier function, immunomodulation and neurotransmitter production [12]. Whether the benefits of probiotics relied on the gut microbiota was still unclear, because some intestinal microbiota did not change after probiotics was given [13] (Table 1).

Adequate amounts of a wide range of micronutrients are needed by body tissues to maintain health. Dietary intake must be sufficient to meet these micronutrient requirements [14]. Essential trace elements like iron, manganese, copper, and zinc play a crucial role in development, growth, and metabolism [15]. They participate in various metabolic processes by acting as cofactors for enzymes or providing structural support for proteins. Deficiency or toxicity of these metals can significantly impact human and animal health, leading to various metabolic and neurological disorders. The gut microbiome affects complex mechanisms of metabolism of essential minerals such as iron, calcium, magnesium, zinc, and selenium [16]. The proper breakdown, absorption, and elimination of these trace metals are tightly regulated processes that require intricate interactions. Crosstalk between the host and these micronutrients is also necessary. The gut, being a complex system, acts as an interface for these components, but other factors contributing to this delicate interaction are not well understood. The gut houses trillions of microorganisms and microbial genes, collectively known as the gut microbiome, which play a role in regulating the metabolism and transport of micronutrients and promote the bioavailability of trace metals either through absorption from food sources or by competing with the host. Additionally, deficiency or toxicity of these metals can influence the intestinal microenvironment, including microbiota, nutrient availability, stress, and immunity. The micronutrient–microbiome axis is bidirectional. On the one hand, microorganisms in the digestive system need micronutrients to develop and operate. The makeup and function of the gut microbiota are significantly influenced by the host’s diet and vita-min supplementation. Specifically, the administration of vitamin A, C, B12, and D supplements leads to modifications in the gut microbiota’s composition by encouraging the colonization of several advantageous species belonging to the *Lactobacillus*, *Bifidobacterium*, and *Roseburia* genera. It has been demonstrated that iron, calcium, zinc, and magnesium supplementation modulate the gut microbiome. Determining the impact of mineral deficit or supplementation on the gut microbiota is an emerging subject [17,18].

Therefore, understanding the role of the gut microbiota in the metabolism of manganese, iron, copper, and zinc, as well as its impact on heavy metal deficiency and toxicity, and vice versa, could open up opportunities to develop improved or alternative therapeutic strategies to address emerging health issues [19].

### 2.2. Fatty Acids and Microbiota

There have been numerous studies exploring the impact of a high-fat diet on the gut microbiota, but many of these studies have not provided detailed information about the specific compositions of the diets and the ratios of fatty acids used. However, some research suggests that the effects of a high-fat diet on the gut microbiota may be influenced by bile salts [20]. Atsushi Yokota and colleagues proposed that bile salts could play a critical role in determining the formation of gut microbiota following high-fat diet treatment. Specifically, cholic acid and its bacterial metabolite, deoxycholic acid, were found to exhibit different bactericidal activities on *Firmicutes* and *Bacteroidetes*, two major bacterial phyla in the gut. This could lead to an imbalance in the ratio of *Firmicutes* to *Bacteroidetes*, which is often observed in animals fed a high-fat diet [21]. This research suggests that bile salts may act as a mediator in the changes observed in the gut microbiota due to high-fat diets, shedding light on a potential mechanism behind the observed alterations. Understanding the interplay between diet, bile salts, and gut microbiota may provide valuable insights into the link between high-fat diets and gut health.

#### 2.2.1. High-Fat Induced Model

High-fat diet (HFD) has been linked to an increased risk of colorectal cancer (CRC) [22]. To investigate the underlying mechanisms, researchers used mouse models of CRC and fed them with either an HFD or a control diet, with or without antibiotic treatment. The gut microbiota and metabolites were analyzed using sequencing and mass spectrometry.

The study revealed that HFD promoted colorectal tumorigenesis in the mouse models. However, when gut microbiota was depleted using antibiotics, tumor formation in HFD-fed mice was reduced. This suggests that the gut microbiota plays a crucial role in HFD-driven CRC development [23]. Further analysis showed that HFD altered the composition of the gut microbiota, favoring the growth of pathogenic bacteria while depleting probiotic bacteria. Additionally, HFD impaired gut barrier function, potentially allowing harmful substances to pass through the gut barrier and interact with the intestinal cells [24]. Furthermore, the transfer of stools from HFD-fed mice to germ-free mice resulted in increased cell proliferation, impaired gut barrier function, and induction of oncogenic gene expression. This indicates that the changes in gut microbiota caused by HFD are capable of promoting CRC development.

The study highlights that HFD drives CRC development by inducing dysbiosis in the gut microbiota, disrupting gut metabolites (e.g., elevated lysophosphatidic acid), and impairing gut barrier function. Understanding the intricate relationship among HFD, microbiome dysbiosis, and metabolite changes is crucial for comprehending the development of colorectal cancer [22].

#### 2.2.2. Polyunsaturated Fatty Acids

It is well established that a ratio of n-3/n-6 fatty acids is beneficial in improving metabolic syndromes induced by a high-fat (SFA) diet. Research by Liu et al. demonstrated that a 1:1 ratio of n-3/n-6 fatty acids had significant positive effects on reducing visceral fat weight, lowering blood lipids, and improving glucose tolerance and insulin sensitivity compared to a 1:4 ratio. This was accompanied by a corresponding down-regulation of inflammatory cytokines and TLR4 protein [25]. In a large cohort study, it was suggested that long-term intake of higher n-3/n-6 polyunsaturated fatty acid (PUFA) can decrease the risk of ulcerative colitis (UC). However, no association was found between total PUFA, monounsaturated fatty acid (MUFA), SFA intake, and the risk of UC. In contrast, trans-unsaturated fatty acids were associated with UC and systemic inflammation [26].

Fatty acids play a role in altering the composition of gut microbiota, and in turn, gut microorganisms can affect fatty acid metabolism. Lactobacilli and bifidobacteria have the ability to generate conjugated linoleic acid (CLA) from the free form of linoleic acid, which is a typical PUFA found in many seed oils [27]. CLA-producing *Bifidobacterium* of human origin, such as B. breve NCIMB 702258, can modulate the fatty acid composition of the host, including significantly elevated concentrations of c9, t11 CLA in the liver. These probiotics also demonstrated significant anti-inflammatory effects on cytokine production, which is relevant to the pro-inflammatory cytokine profile associated with obesity [28].

Overall, these studies highlight the significant influence of fatty acids on gut microbiota composition and metabolism, as well as the reciprocal impact of gut microorganisms on fatty acid metabolism and inflammation. Understanding these interactions may provide valuable insights into developing dietary interventions for various health conditions.

Breastfeeding has a significant impact on the microbiota of infants during the early stages of growth, up to 9 months old. After 9 months, breastfeeding no longer affects the Shannon Weaver index of bacterial diversity. However, when infants stop breastfeeding and are given fish oil supplementation, it leads to significant changes in their microbiota compared to sunflower oil supplementation. Fish oil, which contains n-3 long-chain polyunsaturated fatty acids (LCPUFA), impairs the growth of *Bacteroides* thetaiotaomicron, but not *Escherichia coli*. It also has a major effect on the overall bacterial community and reduces the number of bacterial bands in the gut [29]. Interestingly, despite the potential benefits of n-3 fatty acids on cardiovascular events, an intervention trial found that their beneficial effects were limited [30].

Perilla oil (rich in omega-3 α-linolenic acid) increased the abundance of *Akkermansia muciniphila*, a probiotic associated with reducing the gut barrier disruption and insulin resistance [24,31], while thermally oxidized sunflower oil stimulated the proliferation of *Bifidobacterium*. The study emphasized that long-term frying with vegetable oils rich in PUFAs should be avoided due to the complex effects on gut microbiota [32].

**Figure 1:**
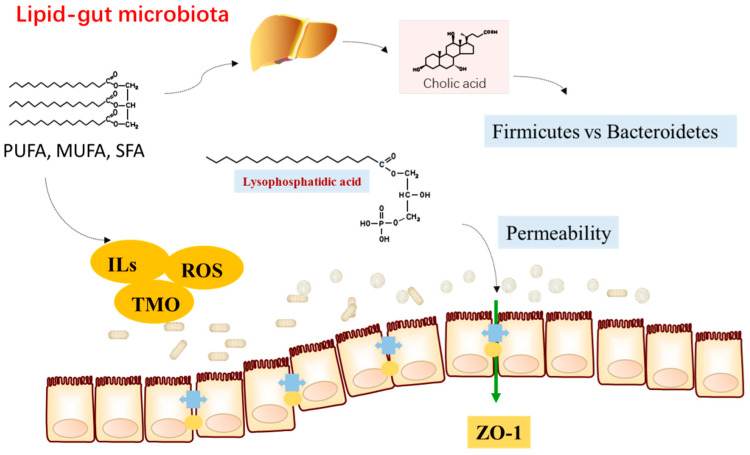
The interaction of dietary lipids and gut microbiota. TMO: Trimethylamine N-oxide, ILS: Inflammatory Lipid Signals, ROS: Reactive Oxygen Species, ZO-1: Zonula Occludens-1.

Obesity is linked to dysbiosis of the gut microbiota and metabolic alterations in adipose tissue. The activation of brown adipose tissue (BAT) and white adipose tissue (WAT) browning has emerged as a therapeutic approach to increase energy expenditure. n-3 PUFA has been found to induce thermogenic effects and restore GM composition. However, the precise role of the gut microbiota in mediating the thermogenic effects of n-3 PUFA requires further research, including clinical trials, to validate these findings and explore the underlying mechanisms [33] (Figure 1).

#### 2.2.3. The Comparison of Different Fatty Acids

Most microbiota analysis has relied on 16S Illumina-based sequencing, which lacks depth in identifying specific bacterial species. However, some studies have utilized different fatty acid compositions while maintaining similar calorie consumption to study the effects of different fatty acids on the gut microbiota. SFA induced an increase in *Firmicutes*/*Bacteroidetes* ratio, and higher weight gain than MUFA and PUFA. MUFA and n-6 PUFA intake was negatively related to the *Bifidobacterium*, but n-3 PUFA was positively related to the *Lactobacillus* group. Many studies indicated that the effects of n-3 PUFA on gut microbiota were not definitely correlated with the body weight control [34]. Different oils have also been shown to influence gut microbiota differently, for example, a palm oil diet reduced the *Bacteroidetes* level, while an olive oil diet and an n-3-rich flaxseed/fish oil diet increased the population of the *Baceteroidaceae* family and *Bifidobacterium* [35].

Conjugated linoleic acids have been found to significantly increase the *Bacteroides*/*Prevotella* ratio and *Akkermansia muciniphila*, but the dose of linoleic acids was higher than the control group in this study [36]. Studies comparing the effects of different types of fatty acids have also been conducted. Saturated fatty acids were found to increase bacterial DNA content in mesenteric fat, particularly *Akkermansia* and members of Lachnospiraceae, suggesting the invasion of bacteria in intestinal epithelial cells. Fish oil treatment decreased the abundance of specific bacterial species like Helicobacter, Uncultured bacterium clone WD2_aaf07d12 (GenBank: EU511712.1), Clostridiales bacterium, Sphingomonadales bacterium, and Pseudomonas species *Firmicutes* [37] (Table 2).

### 2.3. Carbohydrate and Gut Microbiota

#### 2.3.1. Dietary Fiber

Dietary fiber intake plays a crucial role in maintaining a healthy gut microbiota and is linked to metabolic disorders such as obesity and diabetes. The response of the gut microbiota to dietary changes depends on the type, amount, and duration of fiber intake. For example, resistance starch (RS) found in many plant-source foods can increase the abundance of beneficial bacteria like *Faecalibacterium prausnitzii* and propionate-producing microorganisms [44]. It can also decrease potentially pathogenic taxa like *Escherichia coli* and *Pseudomonas* spp. Soluble fiber products obtained from hydrolyzed rye bran and oat fiber residue have been shown to increase the abundance of *Bifidobacteria* and *Lactobacillus*, as well as total SCFA levels in the cecum. A guar diet has been associated with a higher abundance of *Akkermansia muciniphila*-like bacteria compared to a high-fat diet (HFD). In vitro fermentation studies with various fibers and *Lactobacillus acidophilus* NCFM showed that some fibers had no significant effect on the *Bacteroidetes*/*Firmicutes* ratio, but only slightly decreased it. However, the fermentation increased lactic acid content significantly. Studies have also found that different dietary fibers can influence the colonic microbiota composition and SCFA concentrations differently. For instance, RS did not affect colonic microbiota composition, but other fibers like inulin, oligofructose, and guar gum did. High-level dietary fiber treatment stimulated the growth of beneficial bacteria like *Bifidobacterium*, *Lactobacillus*, *Enterococcus* group, and *Ruminococcus* group. Reduced dietary fiber intake and increased consumption of fat, sugar, and animal protein negatively impact gut microbiota and contribute to chronic diseases. Therefore, dietary fiber-rich and wholegrain diets are important for improving the intestinal environment and promoting host health. Future research should focus on the relationship between dietary fiber structure and gut microbiota-mediated host health, the influence of SCFAs on psychological functions, personalized nutrition management, and the effect of food processing on dietary fibers to optimize the relationship between dietary fiber, gut microbiota, and human health.

Dietary fiber is a type of non-digestible carbohydrate that plays a critical role in maintaining the health of the intestines [45]. It is broken down and fermented by the gut microbiota in the large intestine, leading to the production of beneficial compounds like SCFAs and other metabolites [46]. The physiological effects of dietary fiber are diverse and significant, including promoting regular bowel movements, reducing the risk of colorectal cancer, stabilizing blood sugar levels, lowering blood lipid levels, improving inflammation, and exhibiting properties that modulate the immune system and combat tumors [47,48,49,50].

The relationship between dietary fiber and the intestinal microbiota is crucial in understanding its bioavailability and potential health benefits. This knowledge can guide the development of healthy foods, enhance food processing techniques, and improve their applications. By comprehending the complex interplay between dietary fiber and gut microbiota, effective interventions can be developed to address noncommunicable diseases and promote overall health.

Notwithstanding substantial advancements in elucidating the symbiotic dynamics between dietary fiber and gut microbiota in human health, critical lacunae persist within the existing body of knowledge. Thus, rigorous and systematic inquiry is indispensable to dissect the nuanced intricacies underpinning this intricate nexus and to delineate specific loci for intervention aimed at ameliorating noncommunicable diseases with precision. The ongoing scholarly expedition in this domain harbors profound potential to propel our comprehension to unprecedented depths and, in turn, to catalyze paradigm shifts in health outcomes at a global scale.

#### 2.3.2. The Polysaccharides

Polysaccharides are essential bioactive compounds found in various foods and herbal medicines. In traditional Chinese medicine, when preparing decoctions, polysaccharides can be dissolved along with other low-molecular-weight bioactive compounds like triterpenoid saponins, flavonoid glycosides, and alkaloids. The combination of ginseng polysaccharides (GPs) with αPD-1 monoclonal antibody (mAb) has shown promise in enhancing the response to immunotherapy in patients with lung cancer. GPs modulate the gut microbiota, leading to increased antitumor effects of αPD-1 mAb by altering microbial metabolites and T cell populations. Specific bacteria were found to be more abundant in patients responding to anti-PD-1 therapy. Fecal microbiota transplantation from non-responders to mice sensitized their response to PD-1 inhibitors. These findings suggest that the combination of GPs with αPD-1 mAb could improve immunotherapy outcomes, with the gut microbiota potentially serving as a predictive biomarker [51].

Human milk oligosaccharides (HMOs) are abundant in breast milk and are not digested by infants but rather consumed by specific gut bacteria in the lower intestinal tract. *Bifidobacterium* and *Bacteroides* are major bacterial genera in the infant gut that efficiently utilize HMOs. HMOs, acting as potential prebiotics, play a crucial role in modulating the composition of the infant gut microbiota. In vitro studies have shown that HMOs promote the growth of *Bifidobacterium* and *Bacteroides*. Overall, HMOs provide complex carbohydrates as a food source for selected gut bacteria, contributing to the establishment of a healthy gut microbiota in infants [52].

A study examined the microbiota and human milk oligosaccharide (HMO) profiles in breast milk and infant feces from 34 mother–infant pairs. The researchers found that the microbiota composition in foremilk and hindmilk samples was similar, but hindmilk had a higher bacterial load and abundance of oral-associated bacteria. Both milk and feces microbial communities changed significantly over the lactation period. Around 33% and 23% of the bacterial taxa in infant feces were shared with the corresponding mother’s milk at 5 and 9 months of age, respectively. *Streptococcus*, *Veillonella*, and *Bifidobacterium* were among the most frequently shared bacteria. The study suggests that milk-associated bacteria and HMOs play a role in shaping the infant gut microbiota [53]. (Table 3).

### 2.4. Studies on the Potential Prebiotic Effects of Plant Compounds on the Intestinal Microbiota

The systematic review on the impact of dietary protein and its processing on the gut microbiota highlights the importance of factors like protein source, content, composition, and processing in influencing protein fermentation, absorption, and functional properties in the gut. These factors ultimately affect the composition of the gut microbiota and human health. Selecting high-quality protein sources and appropriate processing conditions is crucial for a positive impact on gut microbiota and overall health. However, further research is needed to fully understand the complex relationship between dietary protein and the gut microbiota [68].

Milk products, including lactose-free milk and glycomacropeptide (GMP), have shown prebiotic potential on the gut microbiota of elderly subjects. Lactose-free milk maintained fecal microbiota diversity similar to the control diet, and both milk diets influenced the abundance of health-related bacterial taxa. However, the in vivo prebiotic activity of GMP was not replicated, despite showing prebiotic potential in vitro. These findings suggest that bovine milk, especially lactose-free milk, may have novel prebiotic potential for human nutrition [69].

The administration of bovine lactoferrin increased the *Bacteroidetes*/*Firmicutes* ratio and fecal *Bifidobacterium* spp., while not affecting *Akkermansia* spp. Bovine lactoferrin also decreased pro-inflammatory mediators and serum LPS, while increasing the expression of zo-1, indicating potential protection of the intestine from LPS invasion. Conversely, a high-fat diet was found to impair the cecum and colon barrier by increasing zo-1 expression and LPS contents. In this study, whey protein, especially hydrolyzed whey protein, showed significant effects on the gut microbiota, influencing the ratios of *Firmicutes/Bacteroidetes* and *Proteobacteria phyla*. The role of specific bacteria like *Akkermansia muciniphila* and *Helicobacter* spp. in maintaining a balanced microbiota state is highlighted [70].

Overall, these studies emphasize the importance of dietary factors, including protein sources and processing, on gut microbiota composition and its impact on human health. The findings suggest that selecting appropriate dietary components and processing methods can influence the gut microbiota in ways that support health and prevent disease. However, further research is needed to fully understand the mechanisms underlying these relationships and to optimize dietary choices for gut microbiota health.

## 3. Plant Compounds: Prebiotic Effects and Gut Health

Plant-sourced foods typically contain a variety of secondary metabolites, such as polyphenols. Three main beverages, including tea, cocoa, and coffee, are rich in various polyphenols, such as catechins, chlorogenic acids, and flavanol glycosides.

### 3.1. Tea and Plant Extracts

Raw and ripened pu-erh tea extract (PETe) have shown promising effects on obesity and gut microbiota dysbiosis. In mice with high-fat diet-induced obesity, supplementation of both raw and ripened PETe led to similar anti-obesity effects, including reduced body weight gain, fat accumulation, oxidative injury, and low-grade inflammation, improved glucose tolerance, and regulation of lipid metabolism-related genes. However, the analysis of fecal samples revealed that raw and ripened PETe had different effects on gut microbiota. Raw PETe influenced microbial diversity and the relative abundance of Formicutes and Bacteroidetes, while ripened PETe brought the microbiota composition closer to normal. Ripened PETe notably increased beneficial bacteria like *Bacteroides*, *Alistipes*, and *Akkermansia*, while reducing obesity-associated bacteria such as *Faecalibaculum* and *Erysipelatoclostridium*. This suggests that pu-erh tea, especially ripened pu-erh tea, holds potential as a therapeutic intervention for obesity and related conditions through modulation of the gut microbiota [71].

Fuzhuan tea, a fermented tea from Southwestern China, has been found to significantly increase the abundance of *Lactobacillus johnsonii* and *Lactobacillus* sp., while not affecting the *Bacteroidetes* and Verrucomicrobia [72]. The tea leaves of Fuzhuan tea contain gallic acid as the main phenolic compound, which is degraded by the metabolism of allocated catechins and tannins. Gallic acid has been shown to protect against hepatic steatosis, obesity, hypercholesterolemia, and insulin resistance in mice with high-fat diet-induced non-alcoholic fatty liver disease (NAFLD). This effect is achieved through the reversal of high-fat diet-induced disturbances to choline metabolism and gut-microbiota-associated metabolism [73]. These findings highlight the potential health benefits of Fuzhuan tea and its impact on gut microbiota-related metabolic pathways.

Polyphenols are bioactive compounds present in various plant-sourced foods, such as tea, cocoa, coffee, cranberry, aronia, haskap, bilberry, grape, and bitter melon. Several studies have explored the effects of these polyphenols on gut microbiota composition and related health outcomes.

In a study investigating green tea powder, it was observed that supplementation did not significantly affect the *Akkermansia* genus, although *Akkermansia* was negatively correlated with body fat, periovarian white adipose tissue, and plasma leptin levels [74]. Additionally, high-fat diet-induced obesity led to a reduction in the abundance of *Bacteroidetes*/*Prevotella* spp., but increased the *Firmicutes*/*Bacteroidetes* ratio, *Bifidobacterium* spp., and *C. leptum*. Treatment with instant coffee further altered the gut microbiota composition, increasing the Enterobacteriaceae and *C. septum* [75].

In the context of polyphenol-rich cranberry extract, it was found to increase the relative abundance of *Akkermansia* spp., while reducing intestinal triglyceride content and alleviating intestinal inflammation and oxidative stress [76]. Similarly, polyphenols from aronia, haskap, and bilberry reduced the *Firmicutes*/*Bacteroidetes* ratio and increased fecal mucin and IgA levels compared to a high-fat diet [77]. Grape polyphenols were shown to increase the relative abundance of A. muciniphila and reduce the *Firmicutes/Bacteroidetes* ratio [78]. Conversely, bitter melon formulation reduced the *Firmicutes/Bacteroidetes* ratio, *Bacteroides*, and *Ruminococcus*, which are associated with gut epithelial cells’ sugar absorption and potential weight gain [79].

Fermented Rhizoma Atractylodis Macrocephalae was found to protect intestinal epithelial barrier function in response to LPS insult by enhancing beneficial bacteria like *Bifidobacterium* spp. and *Akkermansia* spp., while reducing the *Firmicutes/Bacteroidetes* ratio, *Bacteriodetes*, and *Firmicutes* [80].

Additionally, a study comparing grape powder, extractable polyphenols (EPs), and non-extractable polyphenols (NEPs) effects on hyperlipidemia, intestinal permeability, and gut microbiota found that EP and EP + NEP inhibited the increase in body fat and white adipose tissue induced by a high-fat diet. EP also enhanced tight junction protein ZO-1 levels and increased short-chain fatty acids (SCFAs) acetate, propionate, and butyrate in cecum contents. The grape powder selectively increased the Coprococcus genus and restored the reduction in cecum mucosal bacterial communities. EP + NEP, on the other hand, suppressed the Ruminococcus genus and Mogibacteriaceae family [81].

Overall, the interaction between polyphenols and gut microbiota plays a crucial role in colonic health and inflammation. Polyphenols can modulate gut microbial composition by decreasing pathogenic microbes and increasing beneficial bacteria, leading to potential anti-inflammatory and anticancer properties that protect against colonic diseases such as colitis and colorectal cancer [82]. Further research is necessary to fully understand the underlying mechanisms and optimize the use of polyphenols for improving gut health and disease prevention.

Oral administration of oat phenolic compounds (OPC) has been found to have beneficial effects on metabolic disorders induced by a high-fat diet. OPC supplementation resulted in reduced weight gain, improved glucose tolerance, lowered lipid levels, mitigated oxidative stress, and decreased adipocyte hypertrophy. Additionally, OPC intake influenced genes related to glycolipid metabolism and reduced chronic inflammation caused by a high-fat diet. Notably, OPC supplementation also had a positive impact on the gut microbiota composition, which had been disrupted by the high-fat diet. It increased the abundance of *Bacteroidetes* and reduced the diversity of *Firmicutes*. These findings indicate the potential of oat polyphenols in managing metabolic disorders by modulating the gut microbiota [83].

Similarly, other food extracts have also shown effects on high-fat diet-induced obesity and gut microbiota composition. Dietary Phaseolus vulgaris extract was found to alleviate serum lipids and liver steatosis. In response to a high-fat diet, there was an increase in the proportion of *Firmicutes* and a decrease in *Bacteroidetes*, *Proteobacteria*, and *Verrucomicrobia*. However, Phaseolus vulgaris extract increased the relative abundance of beneficial bacteria, including *Bifidobacterium*, *Lactobacillus*, and *Akkermansia*, in the gut [84].

These studies demonstrate that dietary interventions with specific plant-derived compounds, such as oat phenolic compounds and Phaseolus vulgaris extract, can have positive effects on metabolic health and gut microbiota composition, providing potential strategies for managing obesity and related metabolic disorders. Further research in this area is crucial to fully understand the mechanisms underlying these effects and to optimize the use of these natural compounds for therapeutic purposes.

### 3.2. Polyphenol Compounds

Resveratrol, a typical food polyphenol, has been found to reverse the negative effects of a high-fat diet on gut microbiota. Specifically, it can restore the reduced levels of beneficial bacteria *Lactobacillus* and *Bifidobacterium* and counteract the decrease in *Enterococcus faecalis* caused by a high-fat diet [85].

Chlorogenic acid (CGA) is another food polyphenol that plays a protective role against high-fat and high-fructose diet-induced cognitive impairment by influencing the microbiota–gut–brain axis. CGA supplementation has shown to prevent obesity, insulin resistance, cognitive–behavioral disturbances, and synaptic dysfunction. It enhances neuroactive ligand–receptor interaction genes, reduces inflammation, and increases the diversity of gut microbiota and SCFA-producing bacteria. CGA also regulates energy metabolism and neurotransmitters. The study highlights the potential of CGA in improving cognitive function through the modulation of gut microbiota and metabolites and suggests it as a promising intervention against cognitive impairment induced by the diet [86].

Research conducted by Wu et al. demonstrated that dietary carnitine can lead to the production of trimethylamine-N-oxide (TMAO), which is associated with promoting atherosclerosis. The study showed a positive correlation between TMAO levels and specific bacteria, such as *Arthrobacter* spp. 71755-122, Robinsoniella psoriasis, *Bacteroides* intestinalis, Clostridium viride, and Clostridium saccharogumia. However, the administration of allicin, a component of garlic, was able to reduce the content of Robinsoniella psoriasis in mice fed with dietary carnitine. This suggests that certain functional foods and their active compounds can have effects on specific bacteria and subsequently influence metabolic syndrome markers [87].

In the case of fermented blueberries extract B, it was found to reduce hypertension in rats induced by L-NAME. However, other types of blueberries extract did not show the same effects. Interestingly, the extract B also resulted in decreased levels of specific bacteria, *Clostridium leptum*, and *Desulfovibrio*. While changes in microbiota composition were observed in normal rats, the anti-hypertensive effects were observed only in the experimental rats. This suggests that the role of microbiota as an intermediate factor between blueberries and hypertension is still not conclusive [88].

These studies highlight the intricate relationship among food polyphenols, gut microbiota, and various health outcomes. The modulation of gut microbiota by these compounds can play a crucial role in their beneficial effects on health, and further research is needed to fully understand the underlying mechanisms and implications for human health.

### 3.3. Plant Polysaccharides

Dietary polysaccharides, high-molecular compounds found in many plant-source foods, are primarily metabolized in the colon where various bacteria reside. Short-chain fatty acids, produced through the metabolism of these polysaccharides by gut bacteria, have been considered as the main mechanism responsible for the associated health benefits [89].

Ginseng, red ginseng, notoginseng, and *Gynostemma pentaphyllum* saponins (500 mg/kg) have been shown to enhance the abundance of beneficial gut bacteria such as *Bacteroides*, *Lactobacillus*, and *Bifidobacterium*. Additionally, the *Bacteroidetes*/*Firmicutes* ratio was elevated after the administration of notoginseng and *Gynostemma pentaphyllum*. *Gynostemma pentaphyllum* saponins exhibited time-dependent effects on increasing *Faecalibacterium prausnitzii*, an important butyrate-producing bacteria [90].

Intake of barley has been associated with reduced levels of LBP and MCP-1 in the circulation and an increase in the abundance of *Bifidobacterium* and *Lactobacillus* in the caecum. Whole-grain barley also increased *Akkermansia* and the caecal pool of succinic acid but decreased the proportion of *Bifidobacterium* and the *Clostridium septum* group [90].

In a study using maize feruloylated oligo- and polysaccharides in mice fed a high-fat diet, half of the mice (F-FOPS) showed an improvement in their metabolic phenotype, while the other half (N-FOPS) did not show any changes. In the effective mice (F-FOPS), there was an increase in Blautia and *Akkermansia* genera, along with higher levels of SCFA (acetate, propionate) [91].

These studies demonstrate the significant impact of dietary polysaccharides and saponins on the gut microbiota composition and associated health outcomes, emphasizing the role of gut bacteria in mediating the benefits of these dietary compounds. Further research in this field may provide insights into the development of personalized dietary interventions for promoting gut health and overall well-being.

## 4. Clinical Trails

The study by Majid et al. investigated the effects of fructo-oligosaccharides (FOS) on the microbiota and short-chain fatty acids (SCFA) in adults receiving enteral nutrition. Although there was no significant improvement in microbiota concentrations with FOS intake, fecal butyrate concentrations were found to be significantly increased [61]. This outcome aligns with earlier research by Mitsuoka et al., which demonstrated the beneficial effects of FOS on intestinal microflora and health outcomes. In their study involving elderly patients, the daily consumption of about 8 g of FOS led to a significant increase in the population of bifidobacteria in feces, about tenfold compared to before the administration. This was coupled with a reduction in the average stool pH, indicating an improvement in the intestinal environment conducive to beneficial bacterial growth. The consistency of stools was also improved among subjects, especially those who had soft stools before the intervention [92].

For healthy individuals, a high-fiber diet was observed to affect low-species-richness microbiota in a stochastic manner, while high-richness microbiota showed more stability. Higher levels of caproate and valerate were positively associated with the proportion of Prevotella, Dorea, and Coprococcus genera, but negatively associated with the proportion of the *Bacteroides* genus in the stool [93]. The relationship between prebiotics and overall human health has been an area of increasing interest in recent years. It is a group of nutrients degraded by the gut microbiota, which can provide nutrients to the gut microbiota, and the degradation products are short-chain fatty acids released into the blood circulation, thus affecting not only the GI tract, but also other distant organs. Fructooligosaccharides and galactooligosaccharides are two major classes of prebiotics that are beneficial to human health. Because fructooligosaccharides and galactooligosaccharides occur naturally in small amounts in foods, scientists are trying to produce prebiotics on an industrial scale [94].

Several clinical trials have suggested that inter-individual variance in response to different diet treatment groups is higher than variance between the groups. In obese men, resistance starch enhanced multiple Ruminococcaceae phylotypes but decreased microbiota diversity, while non-starch polysaccharides increased Lachnospiraceae phylotypes [95]. The proportion of propionate was positively correlated with *Bacteroides*, which was highly decreased in the weight loss diet.

NMR-based metabolomics has been applied in the study of microbiota and soluble fiber for humans, revealing associations between fecal metabolites acetate and propionate and different diets [63]. In a cohort study, higher SCFA contents were observed in obese people compared to healthy participants. *Bacteroides*/Prevotella counts were negatively correlated with fecal total SCFA, while the F:B ratio was positively correlated with fecal total SCFA [96]. The F:B ratio between obese and healthy humans was reported to be controversial, with different studies reporting varied results. Andreas Schwiertz et al. found a lower ratio of *Firmicutes* to *Bacteroidetes* in overweight and obese volunteers or increased *Bacteroidetes* in obese people [97,98]. The role of the F:B ratio in influencing the metabolism of food composition (fiber, FOS) and the efficiency of the obesity microbiome remains complex.

*A. muciniphila* has been considered a key bacterium related to glucose metabolism [99]. In a germ-free study, Enterobacter cloacae B29 was found to increase serum endotoxin load and aggravate inflammation in mice with a high-fat diet [100].

Diet-induced obesity is characterized by an increase in the proportions of *Firmicutes* and a decrease in *Bacteroidetes*, accompanied by a decrease in total bacterial numbers [101]. The use of the antibiotic vancomycin was shown to significantly reduce the proportions of *Firmicutes* and *Bacteroidetes* and increase Proteobacteria, leading to improvements in metabolic abnormalities associated with obesity [102]. The microbiota of obese individuals is distinguished from that of non-obese individuals, with decreased microbiota diversity and a lower ratio of *Bacteroidetes* to *Firmicutes* observed in the obese population [103,104].

The study investigated the relationship between dietary omega-3 polyunsaturated fatty acids (ω-3 PUFAs), gut microbiota, microbial metabolites, and the risk of colorectal adenomas. In a case–control study involving 435 participants, a higher intake of ω-3 PUFAs was associated with an 11–55% reduced risk of colorectal adenomas. This association was found to be influenced by the evenness of the gut bacterial community. Additionally, specific gut bacteria and bile acid metabolites were positively associated with colorectal adenomas. These findings suggest that increasing ω-3 PUFA intake and modifying the gut microbial environment could be potential strategies for reducing the risk of colorectal cancer. The study employed 16S rRNA sequencing and global metabolomics to assess the gut microbiota and metabolite profiles in relation to colorectal adenomas [105].

In a clinical study, it was observed that healthy individuals with a higher-fiber diet had higher levels of butyrate and butyrate-producing bacteria compared to individuals with low fiber intake or those with advanced colorectal adenoma, even though they were also supplemented with high fiber in their food. The beneficial effects of fiber intake were not specifically attributed to any particular type of food fiber, as the mixed fibers included nonstarch polysaccharides and resistant starch from wholegrain cereals, corn, seeds, nuts, wheat, barley, rice, and oats. In the *Firmicutes* phylum, healthy individuals had higher levels of *Eubacterium*, *Roseburia*, and *Clostridium*, but lower levels of *Enterococcus, Streptococcus*, and *Bacteroides* (*Bacteroidetes phylum*) compared to unhealthy individuals with advanced colorectal adenoma or healthy individuals with low fiber intake [106].

In another clinical study, it was indicated that *Prevotella* species were more predominant in most Africans, while *Bacteroides* were more predominant in most Americans. *Butyrate producers* (BcoA), *methane producers* (mcrA), and *hydrogen sulfide producers* (dsrA) were found to be more abundant in native Africans. The concentration of butyrate in native Africans was significantly higher than in African Americans, and it was correlated with the butyrate producers *Clostridium cluster* and *Clostridium cluster XIVa*. Additionally, fecal bile acids (cholic acid, chenodeoxycholic acid, and deoxycholic acid) were found to be increased in African Americans [107].

## 5. Conclusions and Perspectives

### 5.1. Conclusions

1. Reaction to food in gut microbiota composition: after several studies were carried out, it was determined that the vital importance of gut microbiota impact is related to food components.

2. Interactions among diet, microbiota, and disease: Microbiota changes are evident through variations in absorption and metabolism of food, which causes different dysfunctions in the gut microbiome-specific diseases. Consumption of nutrient-rich formula at an early age may be one of the opportunities for timely dietary control, as early gut microbiota plays a role in the regulation of life-long metabolic functions.

3. Gut microbiota and systemic implications: the nutrition patterns of followers of the Western and Mediterranean diets result in different microbial populations in their intestines that are associated with immune system properties.

4. The connection between the brain and gut, given that food interactions most likely have an effect on neurodegenerative diseases, should not be ignored.

5. Possible probiotic benefits of food ingredients: Dairy products may have prebiotic positives in the stomachs of older people because of the presence of some health-promoting microbes in the food. It is well known that some bacteria contribute to sustaining gut health according to this approach.

6. Polyphenols and gut health: significantly, polyphenolic-rich food originating from plants, including tea, chocolate, and coffee, may be responsible for gut flora modifications and lead to a number of health benefits.

### 5.2. Perspectives

Oral administration is a typical route of drug administration due to its safety ad-vantage, suitability, and less expensive nature. Thus, many oral drugs will not be able to reach systemic circulation from the small intestinal absorption sites with their rate control formulations. Gut microbiota, which is composed of microbes from bacteria, archaea, and eukarya, are responsible for altering the bioavailability of oral drugs through various pathways. These mutual effects refer to drug biotransformation, regulation of drug transport molecules, and properties of the gastrointestinal tract. The gut microbial composition is an influential factor in drugs’ uptake and bioavailability, and it would not be prudent to ignore them. The understanding of gastrointestinal microorganisms and oral medicines may lead to personalized diagnosis, setting up drug interaction prevention, and optimal drug delivery system designs. This review gives insight into the microbiota, host, and oral drug relationship not only to inform the areas for further research but also to influence drug bioavailability policymaking. Further research should focus on developing personalized nutrition plans that take into account each person’s unique gut microbiota composition, developing novel probiotic and prebiotic therapies based on a thorough understanding of how specific dietary components affect the gut microbiota, and gaining a deeper understanding of how diet affects the gut microbiota and the mechanisms by which it influences health. Investigating the gut–brain axis may also lead to the development of novel nutritional approaches to the treatment of mental health issues and neurodegenerative disorders. Furthermore, in order to verify the effectiveness of diet-based intervention programs and comprehend their effects on gut microbiota and health, comprehensive clinical studies are required. Knowing how nutrition and gut flora interact with other medical interventions, including cancer therapy, will provide additional assistance in a wholistic strategy for managing illness and wellbeing.

## Figures and Tables

**Table 1:** The common probiotic microbiota and their impact on health/disease.

Lactobacillus Species	Impact on Health/Disease
*L. acidophilus*	Vaginal health
*L. amylovorus*	Lowers cholesterol and improves bowel function
*L. casei*	Enhances immune response and improves digestive problems
*L. crispatus*	Prevention of urinary tract infections
*L. delbrueckii* subsp. *bulgaricus*	lactose digestion
*L. gallinarum*	Chicken Gut Health
*L. gasseri*	Weight loss and improved metabolism
*L. johnsonii*	Inhibits intestinal pathogenic bacteria
*L. paracasei*	Reducing inflammation
*L. plantarum*	Helps digestion
*L. reuteri*	Improve dental health
*L. rhamnosus*	Prevention and treatment of diarrhea
Bifidobacterium species	
*B. adolescentis*	Intestinal health
*B. animalis*	Fighting infections of the digestive system
*B. bifidum*	Improve allergy symptoms
*B. breve*	Good for the intestinal health of infants and young children
*B. infantis*	Relief of Irritable Bowel Syndrome
*B. lactis*	improve digestion
*B. longum*	Lower cholesterol, fight inflammation and stress
Other lactic acid bacteria	
*Enterococcus faecalis*	May become pathogenic
*Enterococcus faecium*	cause infection
*Lactococcus lactis*	Fermentation of dairy products
*Leuconostoc mesenteroides*	Food fermentation
*Pediococcus acidilactici*	Maintaining intestinal balance
*Sporolactobacillus inulinus*	Involved in intestinal fermentation
*Streptococcus thermophilus*	Making yoghurt and cheese
Nonlactic acid bacteria	
*Bacillus cereus* var. *toyoi*	Feed for animals
*Escherichia coli* strain nissle	Probiotics for certain conditions
*Propionibacterium freudenreichii*	Cheese production
*Saccharomyces cerevisiae*	Brewer’s yeast
*Saccharomyces boulardii*	Treatment and prevention of various gastrointestinal diseases

**Table 2:** The correlation of microbiota and obesity induced by high-fat diet.

Subject	HF Diet Composition	Increased	Decreased	References
C57/BL6 mice	58% fat, 25.6% carbohydrates, 16.4% protein	*Verrucomicrobias*, *Proteobacteria*, *Akkermansia*, *Parabacteroides*, *Lactobacillus genus genera*	*Bacteroidetes*, *Erysipelotrichaceae_Incertae_Sedis*	[38]
SIHUMI mice	Lard and corn oil		*Anaerostipes caccae*, *L. plantarum*, *C. butyricum*, *B. producta*	[39]
Wistar rats	45% HF		*Lactobacillus* spp.	[40]
Male apoe^−/−^ mice	heat-treated high-fat	*Firmicutes*, *Clostridiales*, *Allobaculum*, *Allobaculum*	*Bacteroidetes*, *Rikenellaceae*	[41]
Wistar rats	(10% lard, 20% sucrose, 2% cholesterol, 1% bile salt and 67% standard chow)	*Akkermansia*	*Bacteroides*, *Prevotella*, *Escherichia*, *Sutterella*, *Parabacteroides*, *Clostridium*, *Blautia*	[42]
Wistar rats	45% of energy as fat (31.4% saturated fats,			[43]

**Table 3:** The effects of dietary fiber on the microbiota composition and SCFAs.

Fiber Source	Subjects	Up-Graded Microbiota	Down-Graded Microbiota	Increased SCFAs	In Vivo or In Vitro	References
RS type 3 vs. Pregelatinized potato starch (DS)	Pig	*Actinobacteria, Weissella*-like group, *Clostridium cluster* IV, IX, XV, XVI, and XVII, *Mollicutes, Fusobacteria*	*Bacilli* (i.e., *Allofustis, Lactobacillus acidophilus*–like group, *Lactobacillus plantarum*–like group), *Clostridium cluster* XI and XIVa, *Deltaproteobacteria, Gammaproteobacteria*	Total SCFAs, Acetate, propionate, butyrate, valerate	In vivo	[54]
Oat fiber	Mouse	*Bifidobacterium*, *Lactobacillus*		Total SCFAs, Propionic, iso-butyric, butyric, iso-valeric, valeric	In vivo	[55]
Barley husks						
Rye bran		*Bifidobacterium*		Butyric, iso-valeric		
Guar		*Bifidobacterium*, *Akkermansia muciniphila-like bacteria*		Total SCFAs, Propionic, iso-butyric, butyric, iso-valeric, valeric		
Waxy maize	Human fecal microbiota		*Bacteroidetes/Firmicutes ratio*	Lactic acid	In vitro	[56]
Potato fiber			*Bacteroidetes/Firmicutes ratio*	Lactic acid		
Potato lintner			*Bacteroidetes/Firmicutes ratio*	Lactic acid		
inulin	C57BL/6J mice			Total SCFA, Acetate, propionate	In vivo	[57]
oligofructose				Acetate, propionate		
arabinoxylan				Total SCFA, Acetate, propionate		
guar gum				Total SCFA, Acetate, propionate		
RS				propionate		
Cocoa Husk	Pig	*Bacteroides*-*Prevotella, Faecalibacterium prausnitzii*, *Bacteroidetes*, *Bacteroidetes/Firmicutes ratio*	*Firmicutes*, *Lactobacillus*-*Enterococcus, Clostridium histolyticum*		In vivo	[58]
Pectin vs. cellulose	Cat	*Firmicutes*, *Chlorobi, Elusimicrobia*, *Proteobacteria*	*Archaea*		In vivo	[59]
FOS VS. cellulose		*Actinobacteria*				
Pectin	Mouse	Not measured	Not measured	Acetate, propionate	In vivo	[60]
Resistant starch (RS)				Total SCFAs		
Fructo-oligosaccharide						
Cellulose				Lactic acid		
Fructo-oligosaccharides (FOS)—six different dietary, sources of nondigestible carbohydrate (soy polysaccharides, resistant starch, arabic gum, cellulose, inulin and oligofructose)	Human	*Bifidobacteria (n.s.d)*		Butyrate	In vivo	[61]
Pectin	Mouse	*Bacteroidetes*	*Firmicutes/Bacteroidetes*	Acetate, propionate	In vivo	[62]
Polydextrose Fiber	Human			No changes	In vivo	[63]
Pectin		*Actinobacteria, Bifidobacterium*, *Proteobacteria*	*Bacteroides*, *Firmicutes*	Total SCFAs, Acetate	In vitro	[64]
Guar gum		*Bacteroidetes*, *Proteobacteria*	*Bacteroides*, *Firmicutes*	Total SCFAs, Acetate, Propionate		
Inulin		*Actinobacteria*, *Proteobacteria*	*Bacteroidetes*, *Bacteroides*, *Firmicutes*	Total SCFAs, Acetate		
Arabinoxylan		*Bacteroidetes*, *Proteobacteria Bacteroides*	*Firmicutes*	Total SCFAs, Acetate, Propionate		
β-Glucan		*Bacteroides*, *Proteobacteria*	*Firmicutes*	Total SCFAs, Acetate, Propionate		
Resistant starch		*Bifidobacterium*, *adolescentis*, *Proteobacteria*	*Bacteroides, Firmicutes*	Total SCFAs, Acetate, Propionate		
Resistant starch	Human	*Ruminococcus bromii, Bifiodbacterium adolescentis*		Butyrate	In vivo	[60]
Arabinoxylan		*Prevotella*		Total SCFAs		
Inulin		*Faecalibacterium prausnitzii*		Not measured		
Amylose Cornstarch	Human			Butyrate, propionate	In vitro	[65]
Whole-grain barley	Rat	*Verrucomicrobia*, *Deferribacteres*, *Bacteroides*, *Blautia, Clostridium (Lachnospiraceae), Ruminococcus (Lachnospiraceae)*, *Akkermansia*, *Adlercreutzia*	*Firmicutes* *Actinobacteria*, *Firmicutes/Bacteroidetes*, *Parabacteroides*, *Clostridiaceae, Dehalobacterium*, *Oscillopira*, *Ruminococcus*, *Mucispirillum*	Acetic, propionic acid	In vivo	[66]
Barley malt		*Bacteroides*, *Roseburia*, *Coprococcus*, *Lactobacillus*, *Blautia*, *Alphaproteobacteria*	*Parabacteroides*, *Akkermansia*	Butyric acid		
Inulin (Orafti HP) and oligofructose	Rat	*Bacteroidetes*	*Firmicutes*, *Firmicutes/Bacteroidetes*		In vivo	[67]

## Data Availability

No new data were created or analyzed in this study. Data sharing is not applicable to this article.
